# Sustainable Utilization of TCM Resources

**DOI:** 10.1155/2015/613836

**Published:** 2015-05-20

**Authors:** Shilin Chen, Yitao Wang, Zhongzhen Zhao, Christine J. Leon, Robert J. Henry

**Affiliations:** ^1^Institute of Chinese Materia Medica, China Academy of Chinese Medical Sciences, Beijing 100700, China; ^2^Institute of Chinese Medical Sciences, University of Macau, Macau; ^3^School of Chinese Medicine, Hong Kong Baptist University, Kowloon Tong 999077, Hong Kong; ^4^Royal Botanic Gardens, Kew, Richmond, Surrey TW9 3AB, UK; ^5^Queensland Alliance for Agriculture and Food Innovation, University of Queensland, Brisbane, QLD 4027, Australia

Sustainable development aims to maintain utilization of finite resources without exhausting them and meet the demand of present and future generations [1]. Sustainability can only be achieved by balancing production and consumption in a reasonable dimension. It offers a vision of progress that integrates immediate and longer-term objectives. However, the nature of our current resource use endangers their future survival. Moreover, the consequences of our resource use in terms of impacts on the environment may induce serious damage to the environment. Sustainable utilization of natural resources should combine market demand of raw materials, ecological stability, and social benefits [2] and regard them as inseparable and interdependent components in the whole progress of industrial production.

Traditional Chinese Medicine (TCM) has a long history of application and has attracted more and more world-wide attention for its significant effect in prevention and treatment of diseases. Wild medicinal resource is the basis of TCM and accounts for nearly 80% of all the sources of Chinese herbs. With the development of the modern TCM industry and gradual degradation of the natural environment, natural reserves of medicinal resources face a tremendous amount of pressure. Many medicinal plants are at risk of extinction and some adulterants have appeared on the market bringing to attention the need to ensure sustainable utilization of medicinal resources. More and more measures have been taken to guarantee the supply [3] and identification [4–6] as well as quality control [7, 8] and new drug development [9].

In this special issue, a total of 15 papers have been devoted to sustainable utilization of TCM resources. Identification of Radix Astragali using DNA barcoding, DNA barcode-based PCR-RFLP, and diagnostic PCR for authentication of Jinqian Baihua She (*Bungarus Parvus*), using ITS2 to identify* Ferula* species and indirubin-containing medicinal plants, are included. Rapid identification of plants in the Asteraceae with improved RBF-ANN classification models based on MOS-sensor E-nose is also provided.

Introduction and cultivation can supply raw medicinal materials in short supply and play an important role in the sustainable utilization of TCM. Prevention and cure of diseases and insect pests and characteristics of soil microbial community in ginseng cultivation are offered. A systematic review on the production mode of Chinese herbal medicines is also presented. This special issue also reports natural resource monitoring of* Rheum tanguticum* by multilevel remote sensing. Traceability based on chemical two-dimensional barcode for controlling quality in the whole process of the industrial chain of TCM is included. Finally, measuring Agarwood formation ratio quantitatively by fluorescence spectral imaging technique and determining oleanolic and ursolic acids using hyphenated ultrasound-assisted supercritical carbon dioxide extraction and chromatography are introduced.

Readers of this special issue will find not only different identification methods and updated reviews on cultivation, but also some important issues influencing quality control. The industrial chain of TCM, from the production of raw materials to the sale of the finished products, is a complicated multilink process. There are still some key questions to be resolved in the whole process including but not limited to breeding, prevention and cure of diseases and insect pests, quality traceability, exploiting new medicinal resources, and the mechanism of drug metabolism.

*Shilin Chen* *Shilin Chen* *Yitao Wang* *Yitao Wang* *Zhongzhen Zhao* *Zhongzhen Zhao* *Christine J. Leon* *Christine J. Leon* *Robert J. Henry* *Robert J. Henry*

## References

[1] Zhao-Seiler N. (2013). Sustainability of Chinese medicinal herbs: a discussion. *Journal of Chinese Medicine*.

[2] Chen S. L., Su G. Q., Zou J. Q., Huang L. F., Guo B. L., Xiao P. G. (2005). The sustainable development framework of national Chinese medicine resources. *Zhongguo Zhongyao Zazhi*.

[3] Li X., Song J., Wei J., Hu Z., Xie C., Luo G. (2012). Natural Fostering in *Fritillaria cirrhosa*: integrating herbal medicine production with biodiversity conservation. *Acta Pharmaceutica Sinica B*.

[4] Nock C. J., Waters D. L. E., Edwards M. A. (2011). Chloroplast genome sequences from total DNA for plant identification. *Plant Biotechnology Journal*.

[5] Wu M. H., Zhang W., Guo P., Zhao Z. Z. (2014). Identification of seven Zingiberaceous species based on comparative anatomy of microscopic characteristics of seeds. *Chinese Medicine*.

[6] Li X. W., Yang Y., Henry R. J., Rossetto M., Wang Y., Chen S. L. (2015). Plant DNA barcoding: from gene to genome. *Biological Reviews*.

[7] Liang L., Zhao Z. Z., Kang T. G. (2014). Application of microscopy technique and high performance liquid chromatography for quality assessment of *Polygonum multiflorum*Thunb. (Heshouwu). *Pharmacognosy Magazine*.

[8] Song Y. L., Jing W. H., Du G., Yang F. Q., Yan R., Wang Y. T. (2014). Qualitative analysis and enantiospecific determination of angular-type pyranocoumarins in Peucedani Radix using achiral and chiral liquid chromatography coupled with tandem mass spectrometry. *Journal of Chromatography A*.

[9] Wu W.-Y., Hou J.-J., Long H.-L., Yang W.-Z., Liang J., Guo D.-A. (2014). TCM-based new drug discovery and development in China. *Chinese Journal of Natural Medicines*.

